# A large-scale proteomic analysis of human embryonic stem cells

**DOI:** 10.1186/1471-2164-8-478

**Published:** 2007-12-27

**Authors:** Thomas C Schulz, Anna Maria Swistowska, Ying Liu, Andrzej Swistowski, Gail Palmarini, Sandii N Brimble, Eric Sherrer, Allan J Robins, Mahendra S Rao, Xianmin Zeng

**Affiliations:** 1 Novocell Inc., 111 Riverbend Rd, Athens, GA, USA; 2 Buck Institute for Age Research, 8001 Redwood Blvd, Novato, CA, USA; 3 Stem cells and regenerative medicine, Invitrogen Corp, 1610 Faraday Ave, Carlsbad, CA, USA

## Abstract

**Background:**

Much of our current knowledge of the molecular expression profile of human embryonic stem cells (hESCs) is based on transcriptional approaches. These analyses are only partly predictive of protein expression however, and do not shed light on post-translational regulation, leaving a large gap in our knowledge of the biology of pluripotent stem cells.

**Results:**

Here we describe the use of two large-scale western blot assays to identify over 600 proteins expressed in undifferentiated hESCs, and highlight over 40 examples of multiple gel mobility variants, which are suspected protein isoforms and/or post-translational modifications. Twenty-two phosphorylation events in cell signaling molecules, as well as potential new markers of undifferentiated hESCs were also identified. We confirmed the expression of a subset of the identified proteins by immunofluorescence and correlated the expression of transcript and protein for key molecules in active signaling pathways in hESCs. These analyses also indicated that hESCs exhibit several features of polarized epithelia, including expression of tight junction proteins.

**Conclusion:**

Our approach complements proteomic and transcriptional analysis to provide unique information on human pluripotent stem cells, and is a framework for the continued analyses of self-renewal.

## Background

Human embryonic stem cells (hESCs) are pluripotent cells isolated from the inner cell mass of the blastocyst [1]. They can be maintained for prolonged periods in culture and differentiate to representatives of the three germ layers as well as trophoblasts and germ cells. This differentiation potential may be used to model certain aspects of human embryogenesis, including the development and differentiation of pluripotent and other stem cell types during the processes of gastrulation, neurogenesis and organogenesis. Thus, hESCs provide a unique and powerful system to study otherwise intractable aspects of human development. Furthermore, these approaches have the potential to provide differentiated cell types for cell replacement therapies of degenerative disorders such as Parkinson's disease and Type I diabetes [2,3]. Before these cell therapy applications are developed, an understanding of the molecular and cellular mechanisms that drive self-renewal and differentiation is required. Fundamental to this understanding is the elucidation of the transcriptome and proteome of hESCs, using approaches that lay a framework for functional analyses of the unique properties of these cells.

Large-scale gene expression analyses such as microarray, massive parallel signature sequencing (MPSS), expressed sequenced tag (EST) enumeration, and serial analysis of gene expression (SAGE) have been used to compare multiple hESC lines [4-7]; hESCs to germ cell tumors [8]; or to differentiated derivatives in embryoid bodies [9-11] or neural populations [12]. These approaches have highlighted an expanded set of transcripts that mark the pluripotent state [4,13,14], cross-species commonalities in the molecular profile of ESCs [6,12,15], prominent receptors expressed by hESCs [8] and pathways that may play a role in the regulation of pluripotency [16,17]. Nevertheless, cataloguing the cellular transcriptome is only predictive of protein expression and typically does not shed light on post-transcriptional regulation. For example, while tens of thousands of transcripts can be followed simultaneously with SAGE, microarrays and MPSS, these methods do not routinely detect differences in transcript splice variants, or polyadenylation status. These differences may have profound effects on translation, as well as the isoform and function of the protein produced. Finally, numerous post-translational modifications are known to regulate protein function, including enzymatic cleavage, covalent coupling to other molecules, glycosylation, phosphorylation and ubiquitination. These issues all highlight potential shortfalls in our understanding of the hESC proteome.

Several practical approaches for proteomic analyses are currently available, the most established of which is the 2-dimensional (2D) separation of proteins by polyacrylamide gel electrophoresis (PAGE). HPLC-tandem mass spectrometry (HPLC-MS/MS) based technology is rapidly evolving and has recently been used to detect protein expression in multiple cell types. An alternate approach is the recent large-scale adaptation of standard western blotting [18]. In this procedure, a large well is used to separate the sample by PAGE and lanes are created on the membrane containing immobilized protein with the use of a manifold. Compatible combinations of primary antibodies are predetermined, with the criterion of being able to identify proteins that do not co-migrate. Different combinations of primary antibodies are added to each well, with appropriate dilutions of each primary antibody so that expressed proteins are detected in a single condition. The scalability of the system depends on defining suitable combinations of primary antibodies, with up to 1000 antibodies in 200 lanes being used in the largest screens thus far. Detection software is used to identify proteins based on their expected and observed gel mobility. Unlike 2D PAGE and HPLC-MS/MS, large-scale western blotting only identifies proteins for which antibodies are already available. While this is not an appropriate screen for identifying uncharacterized proteins, it greatly simplifies the verification and functional analyses of proteins that are detected. In addition, this approach is highly flexible, and if desired can be focused to particular sets of proteins or protein function, such as cell signaling molecules. Importantly, the foundation of this approach is the large amount of data on individual antibodies, which are already available and characterized in the literature.

More recently, two research groups have conducted proteomic analyses of hESCs using MS [19-22]. In the present study, we used two large-scale western blot systems to examine the expression of > 1000 proteins in hESCs and detected > 600 proteins that were grouped into 18 functional classes. In addition, we identified 42 examples of multiple bands for a single protein, likely to be protein isoforms and/or post-translational modifications, and 22 phosphorylation events in cell signaling molecules. We correlated the expression of members of key active pathways in our transcriptional and proteomic databases and confirmed the validity of this approach. Using these approaches we identified new markers for undifferentiated hESCs and highlighted unrecognized epithelial characteristics of hESCs. Our data confirm the importance of proteomic analyses in complementing transcriptional profiling and provide a framework for continued analyses of the molecular and cellular biology of pluirpotent hESCs.

## Results

### PowerBlot analysis of hESCs

**Figure 1:**
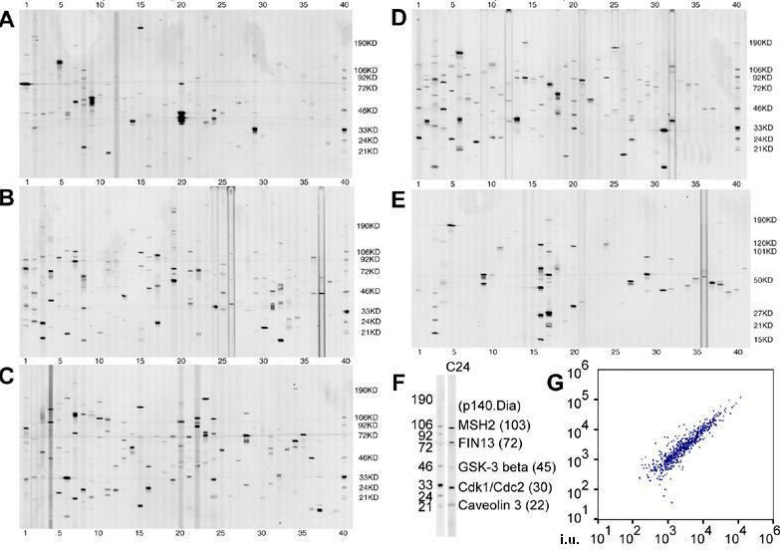
**PowerBlot analysis of undifferentiated BG01 hESCs**. This large-scale western blot consisted of five gels run in duplicate and probed with 934 antibodies. (A-E) One set of blots is shown at a contrast that highlights most bands. (F) A representative lane (gel C, lane 24) aligned with protein markers used for band identification. (G) Scatter-plot of the normalized average intensity (i.u.) values for each protein indicating a linear relationship between duplicate blots. Datasets for this analysis are in Additional Tables 1 and 2.

We first employed a large-scale western blot screen, the PowerBlot system, to profile protein expression in undifferentiated hESCs. This system used 934 antibodies toward proteins representing 22 diverse classes of function, such as transcription factors, the MAP kinase (MAPK) pathway, and apoptosis, among others. To expand a large-scale culture of BG01 cells for this assay, a collagenase- and trypsin- based passaging method was used [23]. While these conditions have been associated with the accumulation of trisomies of chromosomes 12, 17 and X [24], the ease of use of these cultures and similarity in gene expression and differentiation potential to karyotypically normal BG01 hESCs [11,24,25] make them suitable for such large scale applications. For the PowerBlot screen, whole cell lysate from BG01 hESCs was separated on five 4–15% gradient gels. Each blot contained size markers and 39 lanes. Each lane was screened with 1–8 antibodies in combinations that had been predetermined to enable accurate identification of well-separated proteins (Fig. 1A–E). The gels and blots were performed in duplicate and expressed proteins were identified by their predicted size and verified by visual inspection.

A total of 545 antibodies detected bands of appropriate size, which could be compressed to 529 proteins with unique SwissProt identification numbers (Fig. 1A–E and Additional File 1). An enlargement of a representative lane (lane 24 of Blot C) alongside protein markers is shown in Fig. 1F. Thirteen proteins including AKT, caveolin1 and ERK1 were detected in multiple lanes using the same or different antibodies. Information on the antibody catalogue number and dilution, band intensity for each repeat and the averaged value, description of protein function, and Entrez gene and SwissProt database identification numbers is shown in Additional File 1. Three hundred and eighty three antibodies did not detect bands in this screen, indicating lack of expression, or possibly technical issues with detection under standard conditions (Additional File 1).

The size of the detected proteins ranged from 15 kD (GS15) to 280 kD (ABP-280). The average intensity of the detected proteins ranged from 195 to 117926 normalized intensity units (i.u.), with an average of 5367 i.u. The proteins with the highest band intensity were the B2 Bradykinin Receptor (117926 i.u.), Karyopherin α (80698 i.u.), and BiP (74922 i.u.), whilst the proteins with the lowest intensity that could be verified by visual inspection were Inhibitor 2 (247 i.u.), Caspase 8 (201 i.u.), and OXA1Hs (195 i.u.). Finally, the consistency of this assay was demonstrated by plotting the normalized average intensity values for each protein, which revealed a linear relationship between the duplicate samples (Fig. 1G).

### Kinexus analysis of hESCs

**Figure 2:**
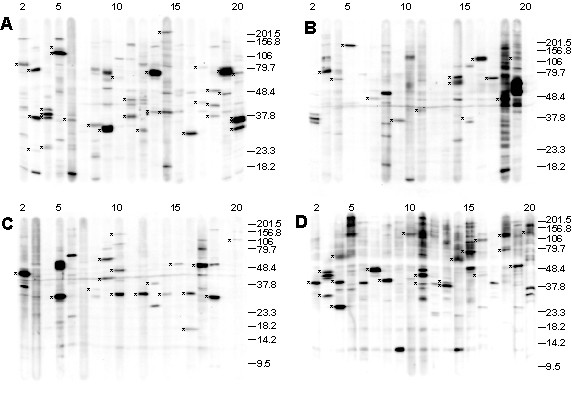
**Kinexus blots of undifferentiated BG03 cells**. Four blots were used to probe BG03 lysate with (A, B) 76 antibodies for protein kinases, (C) 27 antibodies for phosphatases and (D) 37 antibodies for phosphoylated sites in cell signaling molecules. Identified bands are indicated (*). Datasets for this analysis are in Additional Tables 1 and 2.

A more focused screen was used to profile expression of protein kinases, phosphatases and phosphorylated sites in cell signaling molecules in hESCs. The Kinexus assays contained 140 antibodies to these related classes of proteins and phospho-sites. Karyotypically normal BG03 hESCs grown on a fibronectin matrix in MEF-CM [26] were used for this analysis, and whole cell lysate was separated on four 12.5% gels for western blotting. Eighty five immunoreactive bands were identified, representing 38 protein kinases and 16 phosphatases, their isoforms, and 22 phosphorylated sites in signaling molecules (Fig. 2A–D, Additional File 1). Sixty-four antibodies did not detect their corresponding antigen (Additional File 1).

### Functional classification of proteins expressed in hESCs

**Figure 3:**
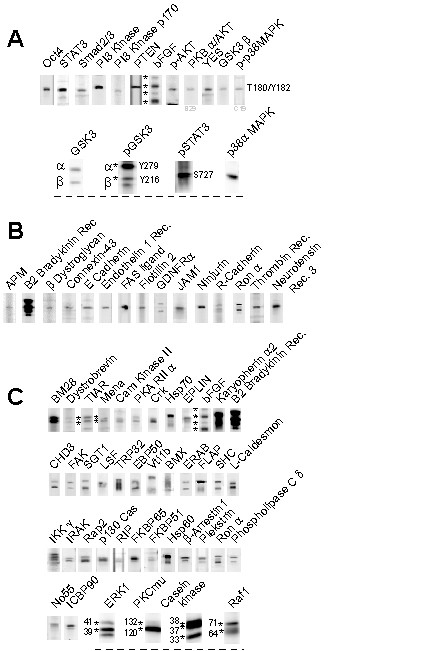
**Functional classification and mobility variants of proteins detected in hESCs**. (A) Proteins with known or suggested roles in self-renewal are shown, including Oct4, STAT3, Smad2/3 and FGF2 (Additional Table 2, "Pluripotency"). Isoforms of FGF2, and phospho-GSK3 are indicated (*). (B) Cell surface proteins are shown, including Connexin 43, E-Cad and GDNFRα (Additional Table 2, "Cell Surface"). Other functional classes of proteins are indicated in Additional Table 2. (C) A total of 42 proteins, including FGF2, HSP70 and ERK1, were found to have multiple bands in either the PowerBlot or Kinexus blots. These bands migrated closely but were sufficiently separated from other detected proteins. Bands predicted to be isoforms of the indicated protein are highlighted in some panels (*).

The PowerBlot and Kinexus assays identified a diverse range of proteins expressed in hESCs. To further annotate these data, the detected proteins were ordered into 18 subgroups based on protein function (Additional File 2). For example, 16 factors with known or implied roles in the regulation of self-renewal or pluripotency of mESCs or hESCs, such as Oct4 [27], STAT3 [28], members of the FGF [29], PI3 kinase [30], Src [31] or MAPK pathways [32], and phosphorylated isoforms of GSK3, STAT3 and p38 MAPK, were grouped under "Pluripotency" (Fig. 3A and Additional File 2). Another functional group (Cell surface) consisted of 20 transmembrane or cell surface proteins (Additional File 2). This included several receptors for peptides and growth factors, such as neurotensin receptor 3, the B2 bradykinin, endothelin 1, and thrombin receptors, and the glial derived neurotrophic factor receptor α (Fig. 3B). These molecules may be useful as targets for cell sorting experiments, and expression of these receptors could identify bioactive peptides or growth factors that may influence hESC self-renewal or differentiation.

Other functional classification of the proteins detected by the PowerBlot screen included: transcription factors (71 proteins), nucleus and nuclear transport (144), cytoskeleton (75), cell adhesion (45), MAP kinase pathway (24), protein kinase A (13), protein kinase C (20), tyrosine kinases (15), adaptors and tyrosine kinase substrates (51), protein phosphatases (17), GTPases and regulators (42), calcium signaling (23), cell cycle (87), apoptosis (61), membrane research (62), and other functions (51) (Additional File 1). Some proteins were included in multiple functional categories due to overlapping properties, such as AIM-1, which was included in the cell cycle as well as in the nucleus/nuclear transport categories. The Kinexus expression data was organized separately into cell signaling-related functional groups (Additional File 1). In addition, 35 proteins were detected by both the PowerBlot and Kinexus systems (Table 1).

**Table 1:** Proteins detected by both PowerBlot and Kinexus systems

**Protein name**	**Swiss Nr**	**Protein name**	**Swiss Nr**
BMX	P51813	MEK2	P36506
CaM Kinase Kinase	Q64572	MKP2	Q62767
Casein Kinase I epsilon	P49674	p38 alpha/SAPK2a	Q16539
Casein Kinase II alpha/CK2a	P19139	Paxillin	P49024
Cdk1/Cdc2	P06493	PKA C	P17612
Cdk5	Q00535	PKC beta	P05771
Cdk7	P50613	PKC delta	Q05655
DAP Kinase	P53355	PP2A Catalytic alpha	P05323
DAP3	P51398	PP5/PPT	P53042
ERK1	Q63538	PTP1B	P18031
ERK2	P27703	PTP1C/SHP1	P29350
FAK	Q00944	PTP1D/SHP2	Q06124
GSK-3 beta	P18266	Rb	P13405
I kappa B alpha	P25963	Rsk	Q15418
IKK beta	O14920	Stat1	A46159
JAK1	P23458	Stat3	P52631
JNK1	P45983	VHR	P51452
MEK1	Q02750		

### Detection of protein isoforms or post-translational variants

Unlike many cDNA-based gene expression assays, western blotting has the capacity to detect multiple protein isoforms due to translation of different mRNA splice variants, as well as post-translational modifications such as enzymatic cleavage, glycosylation, or phosphorylation. Examination of the blots described here identified 42 examples of multiple banding for a single target antigen (Fig. 3C). These candidates exhibited closely migrating multiple bands, which were close to their predicted size but were sufficiently separated from other proteins. For example, four closely migrating bands were observed for FGF2 (Fig. 3C, top panel), which may represent known glycosylation variants of this growth factor [33]. Other known examples of post-translational modifications included those of HSP70, IKKgamma and ERK1.

### Verification of protein expression by immunocytochemistry

**Figure 4:**
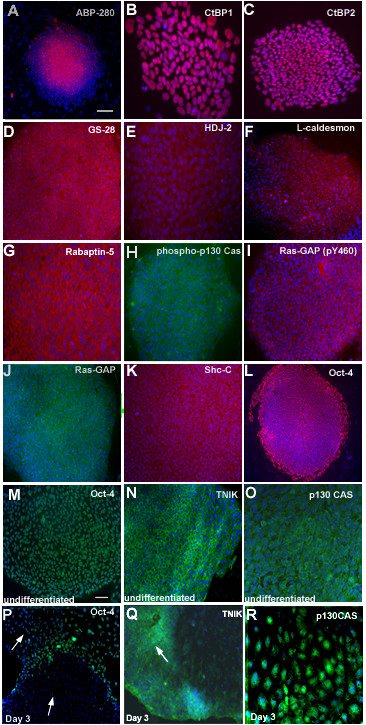
**Verification of protein expression using immunocytochemistry**. (A-K) Ten proteins that were detected in undifferentiated hESCs by western blotting were also detected by immunofluorescence of BG01 cells grown in MEF-CM. Ras-GAP (pY460) is a phosphorylated form of Ras-GAP. The same antibodies were used in this analysis as in the PowerBlot assay, except phospho-p130 Cas (Tyr165). (L) Oct4 was used as a positive control. (M-R) Oct4, TNIK and p130 Cas as markers of undifferentiated hESCs. BG01 cultures were partially differentiated by exposure to 10% fetal bovine serum for 3 days. (M) Oct4 was expressed uniformly in undifferentiated cells, (P) but was downregulated in morphologically differentiated areas after 3 days in serum (arrowhead). (N) TNIK expression was localized to the cytoplasm, and (N, Q) expression appeared to be restricted to morphologically undifferentiated cells (arrowhead). (O) p130 Cas was detected in a membrane/peripheral-cytoplasmic pattern in undifferentiated cells, (R) but this distribution was substantially altered in differentiating cells with a flattened morphology, which exhibited a general cytoplasmic, or perinuclear profile. Scale bar for A-L: (A, L) 200 μm; (C, D, F, H, I, J, K) 100 μm; (B, E, G) 50 μm. Scale bar for M-R: (M, N, P, Q): 100 μm ; (O, R): 50 μm.

The PowerBlot and Kinexus assays identified proteins based on their expected and observed molecular weight, using combinations of antibodies that had been predetermined to detect proteins of sufficiently different sizes. Proteins known to be expressed by hESCs and also identified by these assays, included Oct4, E-CAD, Connexin 43 and Hsp70. To verify expression using a complementary approach, we performed immunoflurorescent staining for 10 proteins not previously reported to be expressed in hESCs by immunocytochemistry, using karyotypically normal BG01 cultures (Fig. 4A–K). These included ABP-280, a homodimeric actin-binding protein often associated with membrane glycoproteins; CtBP1 and CtBP2, two C terminal binding proteins that are a class of transcription corepressors; GS-28, a golgi protein; HDJ-2, a member of the DnaJ-related Hsp40 (heat shock protein 40) subfamily; L-Caldesmon, a cytoplasmic actin-binding protein; Rabaptin, a GTP-binding protein; phosphorylated-p130 Cas, a docking protein with an amino-terminal SH3 domain that may function as a molecular switch that regulates CAS (Crk-associated substrate) tyrosine phosphorylation; Ras-GAP and phosphorylated Ras-GAP (p-Y460), a protein that down-regulates the signal transducer p21^ras^; and ShcC, a protein with an N-terminal phosphotyrosine-binding domain. These proteins were all expressed by hESCs, with the expected subcellular localization (Fig. 4A–K). Oct4 was used as a positive control (Fig. 4L). These results suggested that most of the bands in the PowerBlot and Kinexus assays were likely to be correctly identified.

Preliminary analyses also indicated that expression of some of these proteins was downregulated in differentiated cells, including p130 Cas and the Traf2- and Nck-interacting kinase (TNIK). TNIK is known to be involved in the inhibition of cell spreading via disruption of F-actin [34,35]. Immunofluorescence was used to examine the expression of TNIK and p130 Cas during early differentiation of hESCs. BG01 cultures were partially differentiated by growth in serum containing media for 3 days. This condition generated heterogeneous populations containing Oct4^+ ^cells with characteristic hESC morphology and less tightly packed, and morphologically differentiated areas, lacking expression of Oct4 (Fig 4M, P). TNIK was expressed highly in undifferentiated hESCs, and in the undifferentiated areas at day 3, but was downregulated in areas undergoing morphological differentiation (Fig 4N, Q). This may indicate that TNIK is active in hESCs and degraded rapidly upon differentiation. p130 Cas was detected in a membrane/peripheral-cytoplasmic pattern in hESCs (Fig 4O). The distribution of p130Cas was substantially altered in differentiating cells with a flattened morphology, exhibiting a general cytoplasmic, or perinuclear profile (Fig 4R). This could indicate an alteration in the function of p130 Cas as pluripotent cells differentiate. These analyses suggested that the change in expression or distribution of these proteins could be used as markers for undifferentiated hESCs.

### Comparison of proteomic and transcriptional profiles of hESCs

We have previously employed the Illumina Bead Array system for the large-scale profiling of gene expression in hESCs using 24,000 transcript probes [11]. To compare proteomic and transcriptional analyses of hESCs, the levels of > 600 proteins detected using large scale blotting were correlated with the levels of transcripts detected with the Illumina platform (Additional File 3). In general, a close match between the expression level of transcript and protein was observed: transcripts for nearly all the detected proteins were also identified in the Illumina analysis, and most proteins expressed at high levels also exhibited high mRNA levels.

We reasoned that a focused comparison of specific signaling pathways using a combination of proteomic and transcriptional data was likely to be much more informative than a global interrogation of hESCs. Several major signal pathways that have been suggested to be involved in self-renewal were examined to test this approach. These included the FGF, TGFβ, GSK3β/Wnt/β-catenin and Jak/Stat pathways [17,29,36-39], as well as the more recently suggested MAPK/ERK and Gap junction pathways [32,40]. Correlating transcriptional and proteomic data provided direct confirmation that these pathways were present and likely functional in hESCs (Table 2). For example, FGF2 protein was expressed highly in hESCs and expression of key members of the TGFβ, Wnt, Jak/Stat and Gap junction pathways, namely Stat1, SMADs, GSK3β, β-catenin and Connexin 43, were detected in both transcriptional and proteomic databases.

**Table 2:** Signal pathways that may be active in hESCs

	*Name*	*Protein*	*mRNA*
TGF β	Stat1	++++	++
	PAI-1/SERPINE1	+++	-
	Smad2/3	++	++
	Jun	++	+
	Smad4/DPC4	+	++
	Endoglin	+	-

Wnt	CtBP2	++++	+++
	PP2A Catalytic alpha/PPP2CA	++++	+++
	EBP50/SLC9A3R1	++++	++
	beta-Catenin/Ctnnb1	+++	+
	Cyclin D3/CCND3	++	++
	GSK-3 beta	++	++
	Jun	++	+
	Casein Kinase II alpha/CSNK2A1	++	++

Jak-Stat	Stat1	++++	++
	Crk	+++	+
	Stat3/2	+++	++
	Stat6	+++	++
	PTP1B	+++	++
	JAK1	++	-
	Glucocorticoid R/NR3C1	++	-
	Thrombin Receptor/PAR1/F2R	++	+
	SHPS-1/PTPNS1	++	++
	MCM5	++	+++
	Smad2/3	++	++
	Tyk2	++	++
	Jun	++	+
	Bcl-x/BCL2L1	++	++
	Smad4/DPC4	+	++
	Stat5A	+	+

GPCR	B2 Bradykinin Receptor/BDKRB2	++++	-
	Neurotensin Receptor 3/SORT1	+++	-
	Endopeptidase 3.4.24.16/NLN	++	++
	IP3R-3	++	++
	SHC	+	+++

Gap Junction	Cdk1/Cdc2	++++	++
	GRB2	++++	++
	MEK1/MAP2K1	++++	++
	PKA C	++	-
	PKA RI alpha	++	-
	PKC alpha	++	-
	C-Raf/RAF1	++	++
	ZO-1/TJP1	++	+++
	Connexin-43/GJA1	++	++

IGF	PKC iota	++++	++
	MEK1/MAP2K1	++++	++
	Rsk/RPS6KA1	++++	+
	GRB2	++++	++
	MEK2/MAP2K2	+++	+++
	PI3Kinase/PIK3R1	+++	++
	pan ERK/MAPK1	+++	++
	Crk	+++	+
	eIF-4E	+++	++
	ShcC	+++	-
	PAI-1/SERPINE1	+++	-
	C-Raf	++	++
	SHC	++	+++
	PKC beta/PRKCB1	++	++
	NCK	++	++
	PKB alpha/Akt	++	+
	GSK-3 beta	++	++
	Ercc-1	++	++
	Fatty Acid Synthase/FASN	++	+++
	Jun	++	+
	RAFT1/FRAP	++	++
	PTP1D/SHP2/PTPN11	++	++
	SCAMP1	++	++
	Bcl-x/BCL2L1	++	++
	p70s6k/RPS6KB1	+	-
	PI3-Kinase p170/PIK3C2A	+	+
	PTP1B/PTPN1	+	++
	Dok1/p62dok	+	++
	PI3-Kinase p110 alpha/PIK3CA	+	-

ERBB	EphA4/Sek	++++	-
	ShcC/SHC3	+++	-
	c-erb-B2/ERBB2	++	++
	C-Raf/RAF1	++	++
	SHC/SHC1	++	+++

GDNF	I kappa B epsilon/NFKBIE	++++	++
	GRB2	++++	++
	MEK2	+++	+++
	NCK	+++	++
	C-Raf	++	++
	Ras-GAP/RASA1	++	++
	SHC	++	+++
	GDNFR-alpha/Gfra1	++	-
	Jun	++	+
	IKK beta	++	++
	pan-JNK/SAPK1/MAPK10	++	+
	NBS1/ARTN	+	+
	Dok1/p62dok	+	++

Tight Junction	PTEN	++++	++
	PP2A Catalytic alpha	++++	+++
	PKC iota	++++	++
	Sec8/SEC8L1	+++	++
	beta-Catenin/CTNNB1	+++	+
	CDC42	+++	++
	AF6/MLLT4	+++	++
	PKC alpha	++	-
	Yes	++	++
	Rho/ARHA	++	+++
	ZO-1/TJP1	++	+++
	CASK	++	+
	Symplekin/SYMPK	++	-
	Ras/NRAS	++	++
	Casein Kinase II alpha/CSNK2A1	++	++
	VAP33/VAPA	++	+
	alpha-Catenin/Ctnna1	+	++

MAPK	pan ERK	++++	++
	MEK1	++++	++
	Rsk	++++	++
	ERK2	++++	++
	MEK2/Map2k2	+++	+++
	MST3/STK25	+++	+++
	ERK1	+++	++
	CDC42	+++	++
	C-Raf	++	++
	p38 alpha/SAPK2a	++	-
	G3BP	++	+++
	TFII-I/GTF2IRD1	++	++
	MST1/STK4	++	++
	MKP2/Dusp4	++	++
	Ras	++	++
	Phospho-p38MAPK (T180/Y182)	++	+
	pan-JNK/SAPK1	++	+
	Inhibitor2/PPP1R2	++	++
	ABP-280	++	++++
	14-3-3 epsilon/YWHAE	++	++
	MAPKAPK-5	+	++
	TAO1	+	*
	PBK	+	++
	MKK3b/Map2k3	+	+

Protein expression level: > 10,000: ++++; 5,000–10,000: +++; 1,000–5,000:++; 100–1,000: + mRNA gene expression level: > 5,000:++++; 1,000–5,000: +++; 100–1,000: ++; 30–100: +

*: not included in the gene expression array

**Figure 5:**
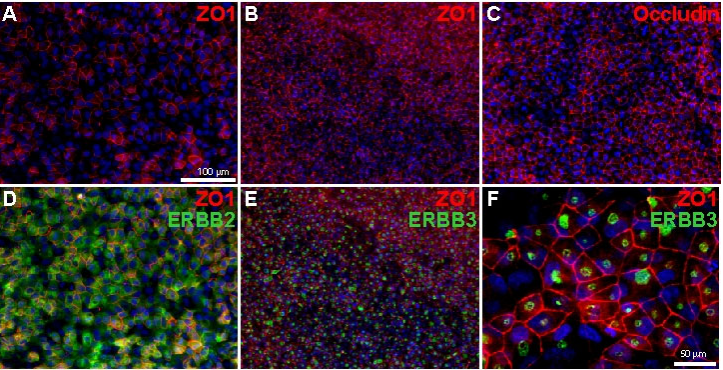
**Tight junction proteins and ERBB2/3 expression in hESCs**. BG01 hESCs were disaggregated to single cells using accutase [52] and cultured in defined conditions. (A) ZO1 expression four and (B) seven days after plating, indicating progressive tight junction formation. (C) Occludin expression 5 days after plating. (D) General cell surface expression of ERBB2, in the same field of view as (A). (E) Localized expression of ERBB3, in the same field of view as (B). (F) Higher magnification of ERBB3 localization in ZO1 expressing BG01 cells, 5 days after plating. Nuclei were stained with DAPI.

This independent confirmation of known networks led us to examine other pathways that showed a similar correlation but have not been identified as key regulators of either self-renewal or differentiation, or suggest unappreciated characteristics of hESCs. Four signaling pathways (IGF, ERBB2, GPCR, and GDNF) and the tight junction complex were highlighted by this analysis (Table 2), and expression of key proteins in these pathways was confirmed. A detailed study demonstrating the importance of the IGF and ERBB2 pathways in hESC self-renewal has been performed and enabled the development of a defined medium for hESC maintenance (TCS and AJR, submitted). Tight junctions are apical cell-cell junctions found in epithelia that establish a barrier to the extracellular environment and a border for apical-basolateral polarity. While hESCs grow in colonies that are highly reminiscent of epithelia, and have been shown to be coupled by gap junctions [40], the formation of tight junction complexes has not been described. hESCs expressed the ZO1 and occludin tight junction proteins along cell borders as expected in polarized epithelia. The distribution of ZO1 expression changed dramatically as hESCs proliferated in culture. When tight junction complexes were disrupted by disaggreagation to single cells, only a subset of cells showed ZO1 staining 4 days after plating (Fig. 5). Continued proliferation to a confluent monolayer on day 7 was accompanied by widespread expression of ZO1, suggesting the formation of a general tight junction barrier. These cultures were undifferentiated and retained uniform expression of Oct4 protein (not shown). ERBB2 and 3 are members of the epidermal growth factor (EGF)-receptor family, which regulate epithelial proliferation via EGF-family ligands. ERBB2 and 3 transcripts are expressed by hESCs [8], are known to function as a heterodimer [41], and transmit a strong proliferative signal for hESCs by Heregulin 1β (an EGF-family ligand) (TCS and AJR, submitted). Immunofluorescence revealed general cell surface expression of ERBB2 on hESCs. Conversely, ERBB3 was highly localized to a concentrated area, and observed in cells that also expressed ZO1. Epithelial cells are known to localize ERBB receptors to the basolateral side of tight junctions, which serves to functionally separate receptors from ligands [42,43]. This is a basic epithelial wound healing mechanism, whereby disruption of the tight junction barrier by injury immediately exposes receptors to extracelluar ligands [43]. These staining patterns are also suggestive of basolateral sorting of ERBB3 in hESCs. The pathways and complexes identified by these analyses lay a framework for future functional analyses of signaling networks in hESCs.

## Discussion

Attempts to harness the potential of hESCs for models of human embryogenesis and cell therapy applications will be greatly enhanced by a detailed understanding of their molecular characteristics. This includes definition of the transcripts, splice variants, and protein isoforms expressed by these cells. Post-translational modifications such as phosphorylation and glycosylation, and the receptors and signaling pathways active in the pluripotent state, or during early differentiation, also need to be determined. This should also be complemented by an understanding of epigenetic characteristics of pluripotency, including methylation, imprinting and chromatin conformation. Such a comprehensive definition of the molecular state of hESCs will enable more accurate prediction and testing of the conditions used for growth and differentiation of hESCs, by precise genetic modification or application of specific growth factor cocktails and reagents. For example, a scalable, fully defined and GMP-certified culture system will need to be developed for the eventual development of hESC-based cellular therapies. Progress has been made in defining growth factor conditions that support self-renewal [44-46], and hESC lines have been isolated in the absence of mouse embryonic fibroblasts and in animal protein free culture conditions [47,48]. A more refined understanding of the biology of hESCs has contributed the development of a defined medium utilizing ligands for IGF1R and ERBB2/3 receptors to promote in self-renewal (TCS and AJR, submitted).

We and others have performed transcriptional analyses of hESCs, using cDNA and oligonucleotide microarrays, SAGE, MPSS and EST enumeration. These techniques have enabled the collation and comparison of transcriptional profiles from multiple hESC lines and their differentiated derivatives and have highlighted an expanded set of hESC specific markers and signaling pathways that may regulate self-renewal or differentiation. Using pathway analysis we were also able to identify key pathways that are active in ESCs (reviewed in [16]). While these efforts have been highly valuable in defining the transcriptional profile of undifferentiated hESCs, they are only predictive of translation and do not shed light on post-translational events in this unique cell type. These processes may also be highly regulated, which could contribute significantly to the overall conversion of genetic information to actual protein function.

We report here a proteomic analysis of pluripotent hESCs by using two large-scale western blotting systems and highlight post-translational events in undifferentiated hESCs. The expression of 545 bands was detected, potentially representing 529 proteins, or their migratory isoforms. In addition, one hundred and forty phospho-specific antibodies were used to identify 85 different phosphorylated sites, on 76 proteins in these cells. The detected proteins were annotated into functional classes representing diverse cellular processes. For example, multiple proteins were detected that have been suggested to regulate the pluirpotent state in mouse ESCs or hESCs. Defining the interplay of these multiple signaling pathways will be critical in understanding the self-renewal versus differentiation decisions of hESCs. Therefore, our data provide a powerful framework for the functional analysis of specific proteins, protein classes, or molecular pathways. In particular, the availability of antibodies for candidate proteins is a major benefit of this approach compared to 2D-gel or HPLC-MS/MS based proteomics.

Although these western blotting approaches are currently more limited in scope than most large-scale cDNA based assays, detecting up to 1000 proteins compared to tens of thousands of transcripts, they have the potential to highlight translational events and post-translational modifications. By comparison, SAGE and MPSS are limited to detecting short sequence "tags" adjacent to the poly-A tail of transcripts, and may not distinguish splice variants with the same 3' exon. We detected 42 proteins with multiple closely migrating bands (Fig. 3C), suggestive of closely related isoforms or post-translational modifications such as phosphorylation. These focused proteomic approaches are therefore likely to be highly complimentary to transcriptional analyses in investigating the functional expression of the genome in hESCs and during cellular differentiation.

One potential issue with this approach is that multiple antibodies are included in each lane, which could possibly lead to misidentification of bands. To demonstrate that identified proteins were expressed in hESCs, the same antibodies used in the PowerBlot assay were used to confirm expression of 10 representative proteins by immunofluorescence (Fig. 4). Furthermore, 13 proteins were detected with multiple different antibodies, and 35 proteins (Table 1) were detected in both the PowerBlot and Kinexus assays. This provided internal, or independent, confirmation of expression of these proteins. Other studies have also demonstrated the expression of several of the proteins we detected in hESCs. These include Oct4, a key marker of the pluripotent state, Connexin 43 and GSK3β, confirming the reliability of large-scale western blotting. Finally, several proteins detected by our assays were also detected in hESCs by MS approaches including Karyopherin α [19].

Additionally, the PowerBlot assay was performed in duplicate, and was shown to be highly reproducible. This suggested that this approach should be informative when comparing hESCs to their differentiated derivatives. Two candidate proteins, TNIK and p130 Cas, were downregulated, or exhibited altered localization upon spontaneous differentiation of hESCs, respectively. This indicated that they were novel markers of undifferentiated cells and molecules that could be functionally involved with self-renewal.

It is impossible in an initial manuscript to analyze and rigorously test all the predictions that could be made from comparing transcriptional and proteomic data sets. However, we did examine key features to illustrate the power of this methodology. Potential new markers for hESCs were identified, the expression and activation of proteins in key self-renewal pathways were confirmed, and a diverse range of proteins were detected and expression correlated with transcriptional analyses. In addition, we highlighted several candidate signaling pathways that may be relevant to self-renewal. Examination of tight junction protein expression indicated that undifferentiated hESCs could form polarized epithelia, which has also been recently suggested by ultrastructural analyses [49]. Discrete localization of ERBB3 may also suggest basolateral separation of this receptor from soluble ligand. These analyses highlight that predictions from a combination of transcriptional and proteomic approaches will serve to focus the investigation of hESCs in the future.

## Conclusion

In summary, we generated a focused proteome of hESCs using large-scale western blotting and sorted the detected proteins according to function and signaling pathways. This characterization provides important basic information on expressed proteins, their isoforms and post-translational modifications, and tools for the continued investigation of the underlying molecular characteristics of hESCs. Importantly, we provide a list of tools, in the form of commercially available antibodies, which can be used to interrogate the function of these molecules in self-renewal or differentiation.

## Methods

### Culture of human embryonic stem cells

For the PowerBlot analysis, enzymatically passaged BG01 hESCs were grown as described previously [23]. These conditions were necessary to scale up the culture to generate the milligram amounts of protein lysate required for this analysis. These conditions maintain cell populations that express the appropriate markers of pluripotency and can differentiate to representatives of all three germ layers, but may lead to eventual accumulation of trisomies for chromosomes 12, 17 or X [26]. For the Kinexus assays, BG03 hESCs were maintained in MEF-conditioned medium (MEF-CM) without the accumulation of karyotypic abnormalities as described previously [14,26].

hESCs were also maintained in a defined medium as indicated. These conditions are described in detail elsewhere (TCS, AJR, submitted). Briefly, the media consisted of DMEM/F12 (Invitrogen), 2% fatty acid-free Cohn's fraction V BSA (Serologicals), 1× nonessential amino acids, 50 U/ml penicillin/streptomycin, 50 μg/ml ascorbic acid, 10 μg/ml bovine transferrin, 0.1 mM β-mecaptoethanol (all from Invitrogen), 1× Trace Elements A, B & C (Mediatech), 10 ng/ml hergulin1β (Peprotech), 10 ng/ml activinA (R&D Systems), 200 ng/ml LR^3^-IGF1 (JRH Biosciences), and 8 ng/ml FGF2 (R&D Systems). Cultures were passaged using Collagenase IV and plated on growth factor depleted Matrigel (BD Biosciences) diluted 1:200. These cultures were karyotypically normal.

To partially differentiate hESC cultures for immunostaining analysis, karyotypically normal BG01 cells were plated on matrigel and grown for three days in DMEM/F12 containing 10% fetal calf serum (HyClone), 1× nonessential amino acids, 20 mM L-glutamine, 50 U/ml penicillin/streptomycin, and 0.1 mM β-mecaptoethanol.

### PowerBlot assays

BG01 hESC lysate was prepared in 10 mM Tris-HCl pH 7.4, 1 mM sodium orthovanadate and 1% SDS, and the PowerBlot assays were performed by BD Biosciences (BD Biosciences). Briefly, 200 μg of protein lysate was loaded in a single, gel-wide well, on a SDS-4–15% gradient polyacrylamide gel. The full PowerBlot screen consisted of five gels, which were blotted and probed with 934 antibodies, and was performed in duplicate with the same cell lysate. The gel dimensions were 130 × 100 × 0.5 mm, and proteins were separated at 150 volts for 1.5 hours, and transferred to an Immobilon-P membrane (Millipore). The membranes were blocked and clamped in a manifold that created 40 lanes across each membrane. A mix of 1 to 8 mouse monoclonal primary antibodies was added to each lane, in dilutions and combinations that had been predetermined to enable accurate identification of well-separated proteins. The predicted sizes of detectable proteins in the blots ranged from 10–540 kD, and the dilutions of the primary antibodies ranged from 1:250 to 1:15,000.

The blots were removed from the manifolds, washed and incubated with goat anti-mouse secondary antibody conjugated to the Alexa680 fluorophore (Molecular Probes). The membranes were scanned using the Odyssey Imaging System (LI-COR). Molecular weight standards were generated by adding a cocktail of antibodies to P190 (190 kD), Adaptin beta (106 kD), STAT-3 (92 kD), PTP1D (72 kD), Mek-2 (46 kD), RACK-1 (36 kD), GRB-2 (24 kD) and Rap2 (21 kD) to lane 40 of gels A-D. Molecular standards for gel E were generated by adding a cocktail of antibodies to Exportin-1/CRM1 (112 kD), MCM (83 kD), Nucleoporin p62 (62 kD), α-tubulin (55 kD), Actin (42 kD), KNP-1/HES1 (28 kD) and NTF2 (15 kD) to lane 16, and antibodies to p190 (190 kD), Hip1R (120 kD), Transportin (101 kD), Calreticulin (60 kD), Arp3 (50 kD), eIF-6 (27 kD) and Rap2 (21 kD) to lane 17.

Bands were detected and raw signal intensity captured automatically using the PDQuest software (Bio-Rad). To normalize the signal intensities, the total raw quantity of each band was divided by the average intensity value of the molecular standards in that image and the normalized values for the duplicate samples were averaged and expressed as normalized intensity units (i.u.). These values represent the relative signal intensity observed for each identified protein band, rather than relative expression levels of different proteins, due to differences in the efficiencies of antibody binding and dilution of the primary antibodies used. Proteins were identified based on the similarity of expected and observed band migration profiles and bands that could not be identified were excluded from the analysis. All identified proteins were verified by visual inspection, and proteins exhibiting a low signal intensity, with an averaged signal of < 1000 i.u., were verified by visual inspection using contrast enhancement in Adobe Photoshop. Bands with > 800 i.u. could typically be observed without additional image enhancement. Microsoft Excel files were generated that contained information on: gel number, lane number, antibody catalogue number (BD Biosciences), protein name, expected size, observed size, repeat 1 i.u. value, repeat 2 i.u. value, averaged i.u. value, antibody dilution, outline of protein function, Entrez gene and SwissProt identification numbers. These tables were used to list expressed proteins (Additional File 1).

### Kinexus assays

Preparation of the BG03 cell lysate and western blotting was performed according to published protocols [50]. Briefly, cell lysate was prepared in 20 mM MOPS pH 7.0, 2 mM EGTA, 5 mM EDTA, 30 mM sodium fluoride, 40 mM β-glycerolphosphate pH 7.2, 20 mM sodium pyrophosphate, 1 mM sodium orthovanadate, 1 mM PMSF, 3 mM benzamidine, 5 μM pepstatin, 10 μM leupeptin, 0.5% nonidet P-40, with the final pH adjusted to 7.2. The Kinexus assays for protein kinases (KPKS-1.2A and B [76 antibodies]), phosphatases (KPPS-1.2 [27 antibodies]) and phosporylated sites in cell signaling molecules (KPSS-3.1 [37 antibodies]) were performed by Kinexus. The Bio-Rad Mini-PROTEAN 3 electrophoresis system was used to separate proteins by SDS-PAGE. For each assay, 250 μg of cell lysate was loaded in a single well spanning the width of the stacking gel, then separated through a 12.5% SDS-Polyacrylamide gel and transferred to a PVDF membrane. A 20-lane manifold was placed over the membrane and a different mixture of up to 3 primary antibodies was added to each well. The combinations of primary antibodies had been predetermined to detect well-separated proteins, avoiding crossreaction to different proteins that co-migrate. The primary antibodies were rabbit and goat polyclonal, and mouse monoclonal antibodies, diluted 1:1000. After incubation with the primary antibodies, the membranes were removed from the manifolds, washed and incubated with a mix of the appropriate secondary antibodies. The secondary antibodies were donkey anti-rabbit (at 1:5000), sheep anti-mouse (at 1:10,000) and bovine anti-goat (at 1:10,000), all conjugated with horse radish peroxidase. The membranes were washed and immunoreactive bands detected by enhanced chemiluminescence (Amersham-Pharmacia) using a FluorS Max Multi-imager (Bio-Rad). Prestained size markers (201.5, 156.8, 106, 79.7, 48.4, 37.8, 23.3, and 18.2 kD) and predetermined human-specific protein migration profiles were used to accurately identify proteins using the Kinexus immuno-reactivity identification system (IRIS) software. Detected proteins were verified by visual inspection.

### Immunocytochemistry

Immunocytochemistry and staining procedures were as described previously [51]. Briefly, cells were fixed with 4% paraformaldehyde for half an hour, blocked in blocking buffer (5% goat serum, 1% BSA, 0.1% Triton X-100) for 1 hour followed by incubation with the primary antibody at 4°C overnight. Appropriately coupled secondary antibodies (Molecular Probes) were used for single and double labeling. All secondary antibodies were tested for cross reactivity and non-specific immunoreactivity. The following antibodies were used: ABP-280 (1:250, BD Biosciences 610798), CtBP1 (1:1000, BD Biosciences 612042), CtBP2 (1:1000, BD Biosciences 612044), GS-28 (1:2000, BD Biosciences 611184), HDJ-2 (1:100, BD Biosciences 611872), L-Caldesmon (1:2000, BD Biosciences 610660), Rabaptin-5 (1:500, BD Biosciences 611080), phospho-p130 Cas (Tyr165) (1:50, Cell Signaling Technology 4015), phospho-Ras-GAP (pY460) (1:250, BD Biosciences 612736), Ras-GAP (1:250, BD Biosciences 610043), Shc-C (1:1000, BD Biosciences 610642), Oct-4 (Santa Cruz biotechnology, 1:200 SC-8628), TNIK (1:100, BD Biosciences, 612250), p130 Cas (1:100, BD Biosciences, 610272), ERBB2 (1:100, Lab Vision, 9G6.10), ERBB3 (1:100, R&D Systems, MAB348), ZO1 (1:100, Invitrogen, 61–7300), or Occludin (1:100, Invitrogen, 71–1500). Hoechst (Invitrogen) or DAPI (Sigma) were used to identify nuclei, and Triton X-100 was omitted when staining for extracellular antigens (ZO1, occludin, ERBB2/3). Images were captured on an Olympus or Nikon fluorescence microscope.

### lllumina data and comparison to proteomic database

Expression levels of proteins detected by the PowerBlot assay were compared to our previous published database of multiple hESC lines examined using the Illumina bead array platform (Liu et al., 2006). Averaged transcript expression signals from the BG01, BG02 and BG03 cell lines were converted to a +/- format, based on the following criteria: A mean transcript detection level of > 5,000 was designated as ++++; 1,000–5,000 as +++; 100–1,000 as ++; 30–100 as +; and signals < 30 was represented as -. In parallel, the protein expression levels were converted to a +/- format based on these criteria: i.u. > 10,000 as ++++; 5,000–10,000 as +++; 1,000–5,000 as ++; 100–1,000 as +. In addition, genes were categorized into the same functional/signaling pathways as per the western blot database.

## Supplementary Material

Additional file 1 Proteins detected by PowerBlot and Kinexus analysis. Click here for file

Additional file 2 Functional classification of proteins detected by Powerblot. Click here for file

Additional file 3 Comparison of proteomic and transcriptional profiles of hESCs. Click here for file
